# The Design Blueprint for a Large-Scale Telehealth Platform

**DOI:** 10.1155/2022/8486508

**Published:** 2022-01-05

**Authors:** Rattakorn Poonsuph

**Affiliations:** School of Applied Statistics, NIDA, Bangkok, Thailand

## Abstract

Technological innovation plays a crucial role in digital healthcare services. A growing number of telehealth platforms are concentrating on using digital tools to improve the quality and availability of care. Virtual care solutions employ not only advanced telehealth technology but also a comprehensive range of healthcare services. As a result, these can reduce patient healthcare costs as well as increase accessibility and convenience. At the same time, the healthcare service provider can leverage healthcare professionals to get a better perspective into the needs of their patients. The objective of this research is to provide a comprehensive design blueprint for a large-scale telehealth platform. Telehealth is the digital healthcare service combining online services and offline access for healthcare facilities to offer various healthcare services directly to patients. This design blueprint covers the digital healthcare ecosystem, new patient journey design for digital health services, telehealth functionality design, and an outline of the platform infrastructure and security design. Ultimately, telehealth platforms establish a completed digital healthcare service and new ecosystem that provides better care for every patient worldwide.

## 1. Introduction

The healthcare industry utilizes digital health services and advanced technologies by deploying multifaceted services for their patients. These tech-enabled services enable patients to monitor and manage their health conveniently. In 2020, Rock Health (see [1]) had conducted a digital health consumer adoption survey that shows consumers interesting in digital health tracking (54%) and live video telemedicine (43%). The growth in consumer adaptation demonstrated by Rock Health has confirmed that digital health services will drive the healthcare industry to reform its business model into fully digital health services.

The global digital healthcare market will reach $510 billion by 2025, growing at a compound annual growth rate of 29.0% from 2020-2025 [2]. The demand for telehealth services has also skyrocketed in responding to the global COVID-19 pandemics. In 2020, a survey from CBInsight [3] from 150 Health-Tech startup companies showed that most startup companies emphasize telemedicine platforms, remote monitoring and diagnostics, covering more than fifty percent of the health technology. Globally, digital healthcare firms have emerged in the global market to take advantage of advancing technologies. Forty-one healthcare unicorns are valued at $102 billion in total [4].

The accelerated growth of telehealth are termed internet hospitals in China; as of July 2020, there are approximately 711 internet hospitals in China, according to research published in the Journal of Medical Internet Research [5]. WeDoctor, China's largest telemedicine network, owns at least 27 internet hospitals and has linked its appointment-making system to 7,800 hospitals across China. The network hosts over 270,000 doctors and has 222 million registered patients. This allows WeDoctor to give users an “integrated online and offline” healthcare experience [6, 7].

Healthcare cost savings, patient convenience, and digital health service integration among participants are the major challenges driving telehealth growth. Towers Watson has reported that telehealth could save at least $6 billion annually to U.S. companies. Currently, health insurance carriers offer telehealth as a regular service in their health insurance coverages and are eligible for reimbursement [8]. However, the healthcare insurance carriers must absorb the medication inflation growth of 8% annually while global GDP growth is only 1.2%. Moreover, the increasing costs of healthcare push consumers with the lowest income to give up their private insurance plan [9]. Emerging global telehealth platforms, the consumer would opt for digital health services because of cheaper healthcare services, the faster response from healthcare providers and more convenience for patients to receive home services. The healthcare service integration across fragmented existing healthcare providers is another challenge to the telehealth platform offering one-stop service to patients without intricate arrangements.

The opportunity to expand a new business model through a new market development is tremendous for telehealth platforms. Healthcare insurance carriers can offer digital health services to an existing customer or new target market with a low-cost operation compared to full service at the hospitals or healthcare facilities. In addition, national healthcare organizations and self-insured employers would benefit most from utilizing a telehealth platform. For example, telehealth triage services have effectively diverted nonacute medical issues from the emergency department to regular routine care. Consequentially, reducing the number of claims will decrease the total claims exposures, leading to lower premiums and subsidiary costs overtime for healthcare insurance carriers.

## 2. Research Concept

During the past decade, the use of the internet to provide digital healthcare services has grown significantly. Therefore, a traditional business related to the healthcare industry needs to adapt by reshaping their health service provision to a digital market, requiring the involvement of multiple services from the multidisciplinary team and stakeholders to provide effective patient care. The telehealth business is recently active with high investment and growth opportunities. An emerging telehealth platform is widespread globally and disrupts traditional healthcare services. The objective of this research is to provide a comprehensive design blueprint for a large-scale telehealth platform. Telehealth is the digital healthcare service combining online services and offline access for healthcare facilities to offer various healthcare services directly to patients. This design blueprint covers the digital healthcare ecosystem and challenges for the telehealth business and digital patient journey design and outlining platform infrastructure and security design.

With existing telehealth platforms, the telehealth design is still in the early stages of a fully functional service among healthcare participants. This research addresses a design of a scalable and efficient telehealth platform that can be extended to cover multiple hospitals and healthcare professionals in the system. In addition, the design blueprint can also be adapted to a broader range of telehealth contexts for a startup business interested in developing and implementing the platform.

## 3. Digital Healthcare Ecosystem

**Figure 1:**
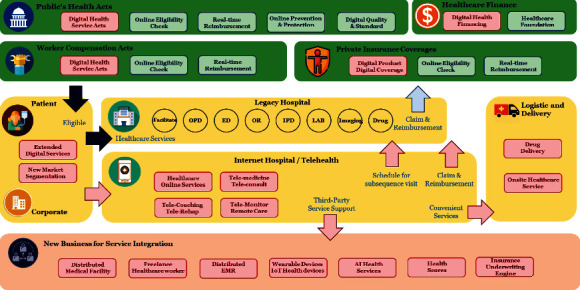
New era of digital healthcare ecosystem.

The digital healthcare ecosystem is associated with several business entities, including the government or national healthcare organizations.  Figure 1 illustrates all participants in the digital healthcare ecosystem. Typically, almost every country provides a primary healthcare service plan under public health policy for citizens and residents. So the healthcare service plan offers benefits to the members by physically accessing healthcare facilities or public hospitals [10]. In addition to the public health policy, some countries provide workers' compensation programs, including medical treatments for work-related injuries or occupational illnesses. However, these healthcare service programs mentioned above offer health services with minimum reimbursement to the healthcare providers and participants. Consequentially, this low-cost program results in the healthcare services at public hospitals or healthcare facilities being overwhelmed with patients, causing long waiting lists and delays of essential medical service.

Digital healthcare services via a telehealth platform are the solution for patients with specific symptoms and chronic health conditions. Patients can stay at the comfort of their own home while receiving a healthcare service with telemedicine from a primary care physician or triage nurse and receive medical delivery [11]. To provide an effective digital health service to their citizens, a government must amend the national healthcare policy to cover digital healthcare services and grant claim reimbursements after using such a telehealth platform. This allows healthcare providers to develop or join these telehealth platforms to offer virtual care to patients. Examples include Medicare and Medicate programs in the US during the COVID-19 pandemic, which allows coverage to a patient using telehealth services to treat certain preapproved conditions and presenting complaints [12].

Meanwhile, the Chinese government has been promoting internet-based medicine since 2014, which issued China's internet-based virtual care legislation framework. In China, the telehealth platform, which is called an internet hospital, is the largest virtual healthcare platform globally [13]. These Chinese internet hospitals collaborate with domestic healthcare institutions along with their registered physicians or as an independent online platform operated by a third-party entity relying on a group of several domestic medical institutions.

Another business participant associated with the healthcare industry is the insurance business, which provides voluntary healthcare coverage products to their clients. Traditional insurance products are similar to the original national health plan requiring patients to access the healthcare facility to gain coverage, even though some healthcare services can be offered virtually via telemedicine [11]. Recently, insurers include telemedicine services into their existing products or introduce innovative products to serve different target customers. Moreover, the opportunities granted from the telehealth platform drive insurers to offer telemedicine and other telehealth services as a new product category with the lower premium to a niche target market segmentation [14].

The telehealth platform offers an integrated service between healthcare providers and patients that were previously not possible. The fragmentation and unconnectedness of existing healthcare services can be integrated into a single application within a digital healthcare service platform [15]. In some countries, healthcare providers are scattered in domestic areas and regions, starting from primary care providers, referring to secondary care, claiming approvals from health insurance carriers, and purchasing drugs from a local pharmacy store. The patient journey is arduous due to the referral process to other specialties, facilities, or hospitals. With telehealth, the combination of previous healthcare services is merged into a one-stop service within the application. The patient can use the telehealth application at home or visit their primary care provider to perform necessary examinations or investigations and virtual consultations with specialist physicians and other healthcare professionals. However, the telehealth platform cannot resolve all of the patient's healthcare concerns. The appointment for subsequent follow-up visits at the hospital is essential for further examination, physical diagnostics, and treatments.

Inevitably, a healthcare provider, a hospital, and a pharmacy store must coordinate with local logistics businesses to deliver drugs and medical supplies to the patient's home. However, in a remote area, the logistics for delivery may be difficult; therefore, the healthcare providers should organize a pickup location for the patients to the nearby pharmacy store or the post office. Moreover, the coordination between healthcare providers and the logistics business will go beyond medical delivery. For example, healthcare IoT products (e.g., portable medical equipment or devices) could be shared and distributed among patients more efficiency. Hence, the logistic partners handle the sharing of the medical equipment by performing borrowing and returning operations.

Healthcare financing is another participant in the healthcare ecosystem that can support people living in poverty having access to healthcare. Initially, healthcare financing forms the foundation that receives the funds from donations to help out a particular group of patients with disabilities. However, the advances in healthcare research and health technology extended healthcare services in multiple places. The particular healthcare treatments include health prevention programs and prolonging wellness treatments, organ transport surgeries, and advanced cosmetics surgeries, which have very high costs for patients and cannot be reimbursed from healthcare insurance carriers. Therefore, healthcare financing can be offered as an affordable program to patients by proposing the healthcare loan directly to the patient via the telehealth platform [16]. Another potential opportunity is the marketing campaign via telehealth for these special treatments with financial assistance.

Telehealth is a digital twin of the hospital since telemedicine was first launched as a digital patient journey and offers ultimate convenience for the patient who can virtually visit the hospital and perform remote consultation with a physician. However, telemedicine is just at the early stages of a comprehensive set of virtual care. Many healthcare services can be offered virtually to the patient, thus reducing the operation loads from healthcare professionals. Telehealth services include viewing and managing an appointment, symptom checker, allergy warning, vital-sign records, electronic health records (EHR), laboratory results, imaging diagnostics results, medical intakes information, and event alerts for patients by using an online application. Besides telemedicine, teleconsulting, telerehabilitation, telepharmacy, and telemonitoring are also included in the telehealth platform [17]. These additional services will be offered to patients as needed based upon individual health conditions.

Telehealth logistics role in the telehealth ecosystem is centered around arranging medical deliveries directly to the patient at home. With telehealth's exponential growth, logistics is essential in ensuring delivery arrangement runs smoothly and is scalable. The challenge of telehealth logistics is to deliver the medicine on a timely basis for the patient and finding a local pharmacy that matches all prescriptions. In case of emergency and the patient is located in a rural area, aviation or drone delivery is required. Telehealth logistics is not only services for medical delivery but also includes shipping of medical supplies or devices, which can be shared among the patients.

Onsite care service is another telehealth service that provides healthcare professionals with the ability to assist the patients in their accommodation. With the onsite service, the telehealth platform offers matching services between patients and caregivers based on the patient's health conditions. As a result, the caregivers can assist the patients onsite with daily routine activities, including accompanying the patient to the hospital, monitoring patient symptoms and health conditions, medication intake assistance, and activities of daily living. Furthermore, the telehealth platform received patient feedback for their satisfaction and comments to improve matching service in the future.

Ultimately, telehealth could create an enormous value chain business model that can integrate with the telehealth platform. For example, the health-spot station is digital kiosks enclosed with a small container, free-standing units that use video consults, and real-time interaction with telehealth devices for remote diagnosis [18, 19]. Another new business opportunity is the virtual care assistant for elderly patients. According to the research from United Nations, which has announced that aged 80 and over are projected to grow to 202 million in 2030 [20]. The virtual care assistant for older adults will be in higher demand, including digital assistant via teleconference, onsite care services, transportation care services, and virtual rehabilitation services. All of these care assistances are integrated into a single platform of telehealth. There is also an opportunity for international healthcare workers to join the telehealth platform. For example, some patients in the US may request telemedicine with a specialist in Chinese traditional medicine and can prescribe the medication from a local Chinese pharmacy around the patient's area. Moreover, the healthcare professionals have no limited to work within their healthcare institution, and they can join the telehealth platform as freelance to earn additional income globally.

These new business opportunities are extended to patient identification, eligibility verification, and secure electronic health record-keeping services. The health and medical records on the telehealth platform, including medical records from all participants, are a crucial element that needs a secure environment to manage and control [21]. In addition, accumulating healthcare information can analyze disease and treatment for medical public health research, find patient insights for healthcare marketing, identify needs for financial services, and calculate a score of the patient risk for the health insurance carrier [22].

Wearable device technology and the internet of things (IoT) are growing exponentially. Advanced features on wearable devices and IoT devices for healthcare directly assist the telemonitoring system on the telehealth platform [23]. The innovation of biosensors combined with the internet of things technology can transmit health information to the telehealth platform in many ways. These valuable technologies enable remote monitoring of patients, including disease prevention and early disease detection. These devices can monitor vital signs such as temperature, heart rate, heart rhythm, breathing rate, and blood pressure. Health professionals who join the telehealth platform can monitor the patient remotely and promptly alert when a patient's condition deteriorates or early onset of an illness. The telemonitor is an essential part of the telehealth services both in remote monitoring patient conditions and worth for data analytics, diagnostic prediction, and insight for a critical alert pattern [24, 25].

The artificial intelligence (AI) health service is another telehealth service that can plug into the platform. AI is the norm for a primary assistant on the telehealth platform. The AI assistant can start from an AI chatbot to ask for the patient's symptoms before making the online appointment or triage illness to direct the patient to the appropriate physician. Advanced AI is possibly substituting physician diagnostics. For example, Ping An Good Doctor is a Chinese startup company providing an AI-supported one-stop healthcare ecosystem platform in China. The solution enables patients to get medical advice, first by a triage with an AI-supported bot that collects their medical history and provides preliminary diagnostic suggestions [26–28].

Digital health engagements motivate patients to achieve and maintain healthy lifestyle habits and prolong patients' life. The health engagements encourage the patient to get involved with the platform by offering rewards, activities, or competition. In addition, a patient may request personalized health coaching based on an AI automated coach for lifestyle navigators. The combination of healthcare information from past medical records on the platform, health tracking from wearable devices, vital signals from IoT devices, and digital health engagement can produce health risk quantification as a health score [22]. The health score of the patient is a valuable outcome to other businesses as well. Several business sectors could use the health score to qualify customer engagement. Health score enables insurers and health institutions to analyze their member's health risks, including automatic pricing engines, accelerated underwriting, and optimized care management. Health score offers patients insight about their health for healthcare providers and partners to match products and services for individual needs. The health score is also valuable for the financial business sector in determining the ability to pay off the loan based on the borrower's health behavior and lifestyle.

In conclusion, telehealth service changes the healthcare ecosystem into a new paradigm with numerous digital healthcare business elements. The telehealth ecosystem also identifies all players in digital healthcare services. Ultimately, thus, the telehealth platform becomes the core infrastructure to integrate all health services into a one-stop healthcare ecosystem to serve the patient better.

## 4. Patient Journey Design

**Figure 2:**
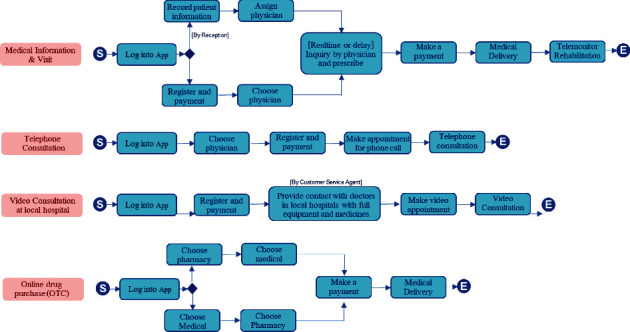
Existing telehealth patient journey pattern.

Telehealth represents an opportunity to redesign the way healthcare services are delivered to patients, allowing patients more accessibility and convenience with a virtual consultation from a healthcare professional. However, existing telehealth platforms offer a simple patient journey, serving only the fundamental medical consultation services.  Figure 2 shows the current existing patient journey pattern schema found in several telemedicine applications.

The main focus of these telemedicine applications is to provide a rudimentary digital health service for patients. These simplified journeys lead patients to achieve only virtual consultation. Four standard services of telemedicine application are the following:

- Providing medical information and virtual visits via telemedicine application by self-service or handling by reception
- Providing regular telephone consultations by appointment
- Providing video consultations at the local healthcare facility
- Offering online drug refills and medical purchases from local pharmacies

In order to improve the virtual healthcare services, similarly to hospitals, the patient journey design of a telehealth platform should have the ability to manage patients with more acute conditions, scalability management, and optimized physician workload. This enhanced version of the patient journey aims to optimize telemedicine services by providing a triage service for patients and integrating it with the existing hospital services where it be specialist input from physicians, nurses, physiotherapist etc. Scalability management is measured by how well the telehealth platform handles volatility regarding the high volume of the patients by evenly distributing the workload to achieve optimal clinical outcomes and avoid excessively lengthy waiting times. In addition, the platform must automatically determine the movement flow of each patient's journey to the examination path differently based on the patient's underlying chronic condition and presenting complaint. The most crucial element is physician utilization, enabling efficient prioritization of work, minimizing idle times and distributing the load to other supporting roles (e.g., teletriage, telepharmacy) where possible.

**Figure 3:**
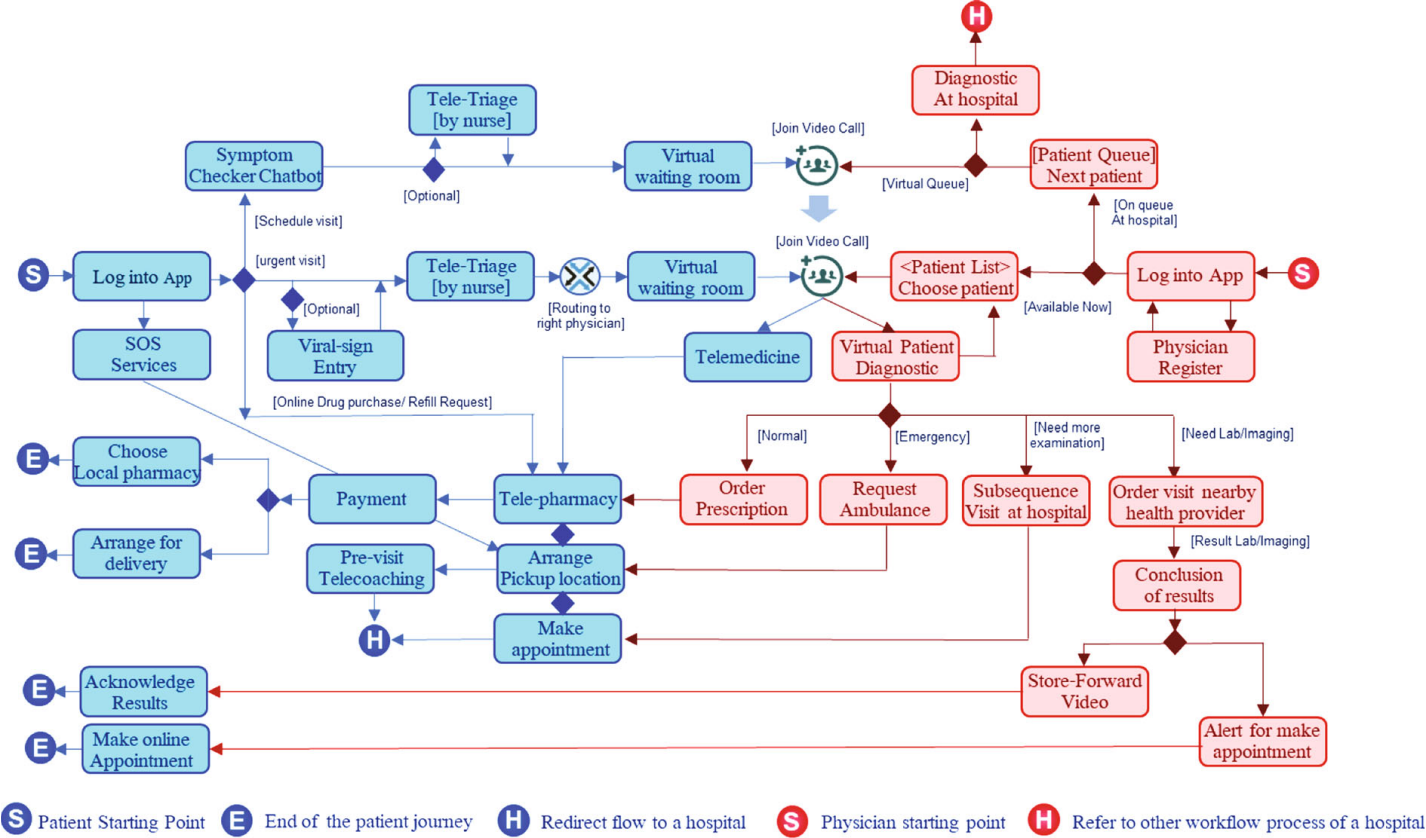
A comprehensive patient journey design of telemedicine services.

Figure 3 illustrates a new comprehensive patient journey design of the telehealth platform. The flow starts from logging into the telehealth application and choosing one for the following services: an “emergency service (SOS),” urgent telemedicine, regular telemedicine, online drug refill, and online drug purchase (OTC). First, the flow for emergency service is to identify the patients' location and then provide previsit instructions. In addition, telecoaching via healthcare professional may be used to supervise patient safety instructions with real-time communication during transit to the hospital.

The flow of urgent telemedicine is treated as an urgent care service. First, the patient will be directly connected to a triage nurse. The triage nurse will then determine the most suitable on-call physician that patient should be connected to, for further diagnosis and management. The patient would then enter a virtual waiting room and waits for the physician to join while the telehealth platform introduces the preparation guidelines. While on the service provider side, after a physician logs into the telehealth application and chooses the “available now” option. The physician can then view “urgent patient list” that are in the waiting room with their corresponding presenting complaints. The physician would have ability to choose to consult patients either in order of severity and or registration/waiting times. At this stage, the telehealth platform can manage concurrent incoming teleconference sessions for both patients and physicians on the global urgent patient waiting list.

The flow of regular telemedicine services is suitable for a regular visit. To minimize healthcare resources on the platform, introducing an intelligent chatbot as the text-first approach is recommended. The intelligent chatbot will help reduce routine task workloads while providing an immediately interactive response with patients. The intelligent chatbot can be used throughout the patient journey within the telehealth platform. Firstly, a symptom checker chatbot is the front-end service after a patient joins a regular telemedicine service. Preliminary history taking can be performed by the chatbot whilst the patient is in the “waiting room,” enabling the physician to then narrow down his/her investigations and diagnostics, early on.

The symptom chatbot can utilize an expert system for automatically matching the patient with a physician based on information from patient interactions. With exceptional scenarios, the symptom checker chatbot will be routed to a triage nurse for virtual consultation if the patient has more complicated conditions before assigning the physician as in urgent telemedicine services and following the same path of navigational flow.

Physician navigation flow on the telehealth platform must be closely coordinated with that of patients. The physicians can choose between two principal roles: exclusively dedicated to treating telemedicine patients and another to treating both telemedicine patients and seeing patients in person in the outpatient department. Most urgent patients get into a flow to meet with dedicated physicians, while nonurgent appointments can meet with dedicated physicians in the telemedicine platform or physicians at the outpatient department, depending on the availability.

After diagnostics, physicians can advise for treatment and prescribe medications to patients for home delivery. In addition, physicians may request the patient to make a subsequent follow-up visit to the hospital. The physician may wish to perform further investigations, So physicians can also ask a patient to stop by a nearby healthcare facility for extra laboratory testing or imaging services, with minimal micro-management involved from the physician.

However, the telehealth platform can handle certain healthcare services partially, while some services require a patient to visit the hospital for physical examinations, investigations, and medical treatments.

Since telehealth is a digital twin of a group of hospitals, the coexistence of the telehealth and the hospital group forms the cross-referral healthcare services. In some circumstances, telehealth is considered as the front-end healthcare service for the hospital group.

This new patient journey design also covers the interchangeability of referral of patients between the telehealth platform and hospital facility.

**Figure 4:**
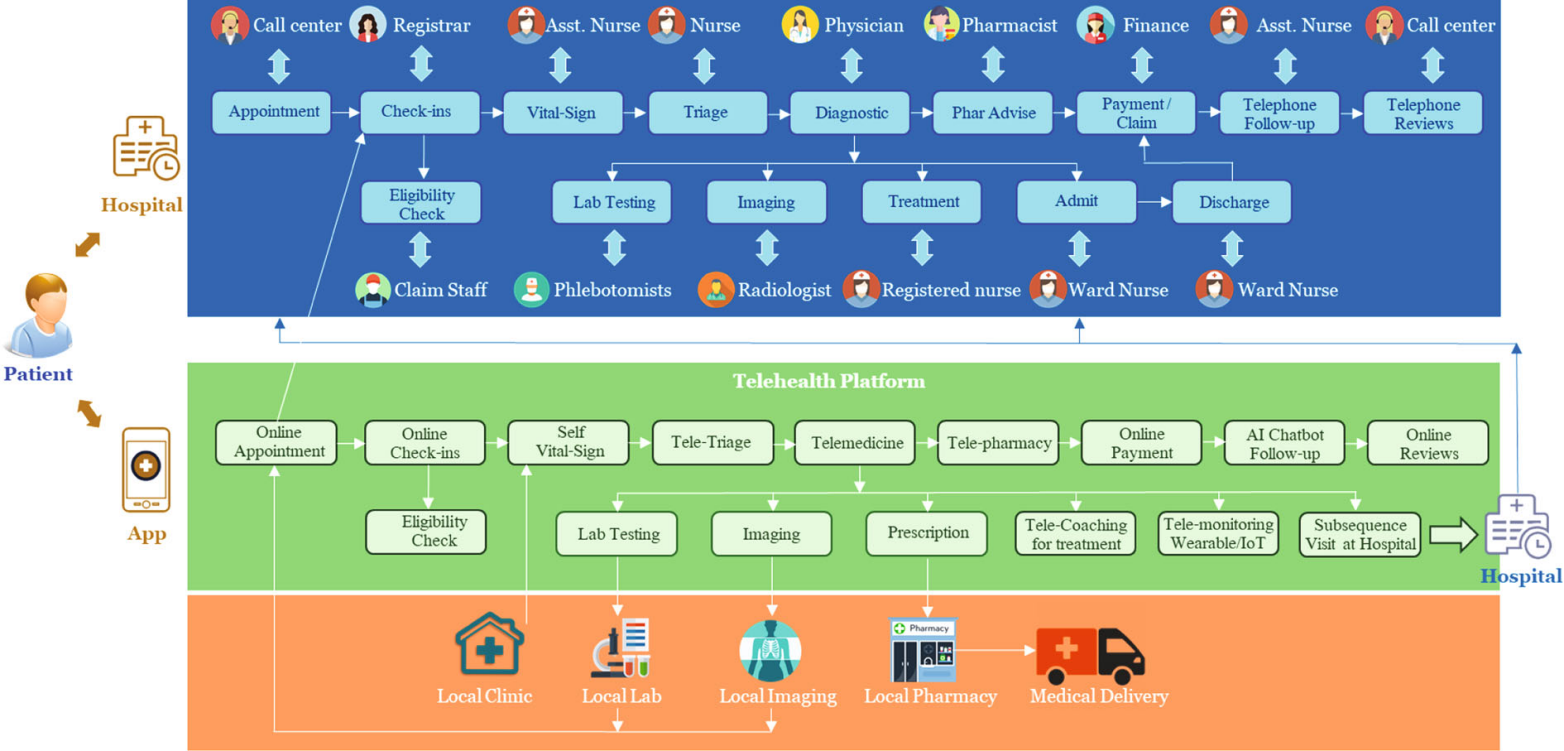
A hybrid design of patient journey.

Figure 4 shows patient journeys that allow patients to choose between digital and traditional channels, which can be interchangeable.

A regular hospital service allows the patient to walk in the hospital with or without an appointment and pass through to the traditional hospital process shown in the top portion in Figure 4, while the patient can make an appointment with a telehealth platform, then choose to proceed with the virtual healthcare service or directly visit the hospital facility.

The patients can choose the digital channel within the telehealth platform. Within the telehealth platform, the patient journey starts from online check-ins and vital-sign measurements. Then, a patient recommends passing through three virtual consultations with teletriage telemedicine and telepharmacist. Finally, the patient makes online payments and then waits for medication delivery at home.

In some circumstances, the physician may also request telemonitoring services. So, the patient is asked to wear a wearable cardiac monitoring device or use an IoT medical device for telemonitoring.

## 5. Telehealth Functionality Design

Typically, telemedicine provides a minimum functionality that covers simplified patient journeys for a virtual consultation with a physician. However, telehealth offers fully digital healthcare services with more functionality than telemedicine. Moreover, with the advance in technologies and telecommunications, some possible conventional healthcare services at the hospital can transform to serve digitally using teleconference along with remote medical devices. Thus, patients get more accessibility and conveniences using telehealth platforms with the extending digital healthcare service functionality.

**Figure 5:**
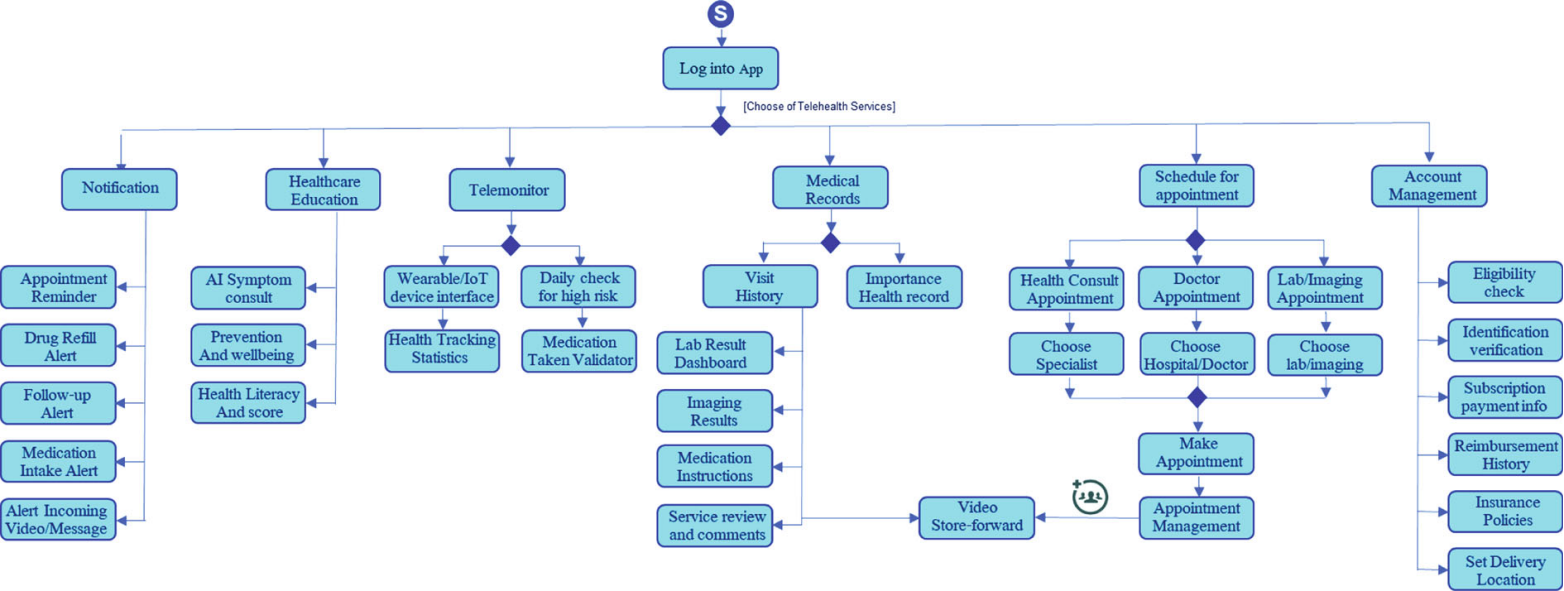
A design of base telehealth functionality.

Telehealth is a future digital healthcare services platform designed to serve patient needs covering potential digital healthcare services similar to visiting the hospital.  Figure 5 illustrates the base telehealth functionality. The telehealth platform provides the following:

- Alert notification
- Healthcare education
- Telemonitoring services
- History of patient's medical record
- Schedule an appointment
- User account management
- Request for telemedicine services

The alert notification is a text-first approach to communicate with a patient. The text-based alert message is an efficient way for a healthcare provider to communicate with patients regarding scheduling appointments, previsit instructions, health campaigns, and promotions. In addition, this can be used in the daily life routine, including medication intake reminders, drug refill alerts, and routine health exercise notifications.

Healthcare education provides a knowledge base for the patient to learn and change to better health. The telehealth platform understands patient insights using advanced artificial intelligence, seamlessly offering health education related to individual interests, including health prevention and prolonged wellness. The health education in telehealth platform is multimedia interactive learning controlled by an expert system. Patients get involved by clarifying the symptoms by answering questions, and the expert system presents healthcare education based upon those facts. With the patient interaction and frequency of use of the healthcare education, the platform can evaluate the patient health literacy and health risk score by using a history of learning.

Telemonitoring is a crucial part of the telehealth platform. Emerging wearable devices, biological sensors, and the internet of things (IoT) for healthcare establish a new way to monitor a patient without being at the healthcare facility [24]. The information flow from those biological sensors will feed to the telehealth platform. Therefore, individual patient health data from the biological sensors will be collected into the platform. As a result, patients can view their health statistics and monitor their health conditions while healthcare providers offer monitoring services. In addition, the telemonitor also includes a daily routine checklist. Patients will get a notification to perform routine health checks such as measuring vital signs, blood sugar level, exercise, or physical therapies, then enter the results of routine health checks into the platform. Therefore, healthcare providers can evaluate the results of routine health checks to further patient's advice. Moreover, the medical-taken validators calculated how many medication doses patients have taken to validate the consistency and limitation of the medicine taken. For example, if the patient takes antibiotics for more than fourteen days, the platform will be warning the patient what can harm the patient via healthcare education.

Appointment scheduling is also an essential feature of the telehealth platform. For chronic illness, the appointment scheduling is straightforward with the same physician to follow up on the patient's health conditions. On the other hand, the emergency request or new appointment is more complicated to find suitable healthcare professionals that match individual patient conditions. Some telehealth platforms offer the patients to search for a particular hospital or healthcare professionals by themselves. Meanwhile, some telehealth platforms provide automatics matching patients' health conditions and preferences with the healthcare specialist.

The medical record is the most frequent use by patients and by healthcare providers. The medical record keeps examination evidence (e.g., laboratory results, X-ray, ultrasound, and ECG) and medication orders from the physician. In addition to the medical record, medication usage instruction, side-effect reviews, and physician comments are also kept in the medical record. Besides, the patient acts as the owner of their medical record and grants the right to access and manipulate their medical records to physicians and related healthcare professionals. The telehealth platform offers an outstanding feature called a store-and-forward video that patients or healthcare professionals could be recording the video clip and send to each other by asynchronous message. The feature enhances the communication among patients, healthcare professionals, and relevant participants in the telehealth platform.

Another mandatory function in the telehealth platform is patient account management, which requires patients to identify themselves with the correct identification, eligibility from the health insurance carriers, patient subscription period, d payment information, other related information for the patient profile.

In summary, the telehealth service functionality is designed to maximize digital healthcare services similar to visiting a hospital. Therefore, the design allows the patient to get better healthcare services with more conveniences. Moreover, the design extends to multiple and cross healthcare providers, which will explain in the next section.

## 6. Telehealth Platform Infrastructure Design

The telehealth platform needs coordination among healthcare providers and relevant participants in the telehealth ecosystem to serve enormous patients. So, the platform infrastructure design must be flexible enough to cover all functionalities mentioned in the previous section. The telehealth platform design is more complicated than a regular matching supply and demand (e.g., Uber: ride-hailing, Amazon: marketplaces on e-commerce, AirBnB: accommodation matching platform) [29]. Unlike single-step demand and supply matching, the telehealth platform naturally has multiple-step demand-supply matching tiers with various life risk factors.

**Figure 6:**
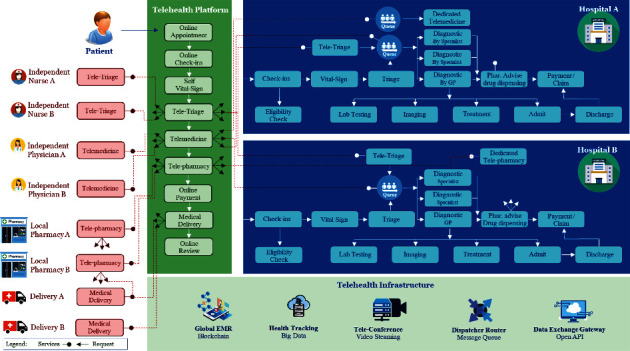
Infrastructure design for a large-scale telehealth platform.

Figure 6 illustrates the infrastructure design of the large-scale telehealth platform. The design allows participants from multiple healthcare providers, independent healthcare professionals, and other participants in the digital health ecosystem to join the telehealth platform. In a competitive environment, each healthcare institution has its strength to serve the patient with its well-designed workflow process to optimize their patient journey. Therefore, each hospital has its workflow process design and has different structures of services. The most challenging design and implementation are integrating various hospital workflow processes into a single streamlined workflow process for patients inside the telehealth platform. The design is a real-time coordinating platform that combines all digital health participants to serve patients globally and digitally.

In addition, some healthcare providers (hospitals) may provide dedicated telemedicine services, while some share the physicians and specialists with the standard services at the hospital. Therefore, the design of telehealth must accommodate various healthcare organization workflow processes and managing flow to serve patients without interruption.

As explained in the previous section, the effective telehealth service and the suitable patient journey design recommended at least three stages of teleconference: teletriage, telemedicine, and telepharmacy for each patient virtual visit. Thus, infrastructure design provides the automatic intelligent call dispatcher to deliver patients with the right healthcare professionals promptly. Meanwhile, the healthcare providers and independent participants should provide the end-point services to the call dispatcher making a request. Once a patient needs a teleconference, the call dispatcher finds available end-point services from the healthcare providers or independent participants. Then initialize of the teleconference is merged from both ends. This automated call dispatcher is an intelligence program that finding the best match of available healthcare professionals based on multiple life risk constraints. The end-point service is virtual healthcare services that are physically distributing throughout the region. This routing process of intelligent call dispatchers obtains information from the participant status (e.g., available, ready to serve, queuing, busy with a patient) and patient health condition (e.g., severity, critical, normal, routine). So, the intelligent call dispatcher routes a patient to a ready healthcare professional or having a minimum of waiting physician's queue. This routing algorithm is overseas the available end-point services from multiple healthcare providers and independent participants in the telehealth platform, which can connect a patient across a healthcare provider's boundary. The automatic call dispatcher also handles all incoming traffic of all patients to the three stages of the teleconference. In each step of the teleconference, participants accumulate health data from patients to use as information for the next dispatcher for routing decisions. However, healthcare professionals can ignore these automated dispatcher services by manually redirecting patients to the appropriate specialist.

The large-scale telehealth platform may integrate with thousands of healthcare providers and independent participants. So, the electronic health record (EHR) is not suitable for storing in the centralized database because of the reoccurring updates from multiple healthcare providers and participants. EHR must be synchronized among local hospital information systems (HIS). Therefore, blockchain technology is more proper for use as a distributed ledger technology for record-keeping of EHR [21]. Blockchain allows all participant database synchronizations as one piece of information while providing high-level security to protect the malicious update from untrusted sources. With enhanced encryption on the blockchain, patient data privacy could be preserved for relevant participants only. A telehealth platform retains blockchain EHR as core data to interface with all participant HIS system. Any update on blockchain EHR in the telehealth platform will replicate and update to participant HIS system too. The feature reduces double entry efforts of the healthcare provider and maintains system integrity.

This research has evaluated four blockchain technologies that are appropriate to keep an electronic health record (EHR). The comparison of blockchain technologies is performed by setting up the blockchain environment with multiple nodes and writing a smart-contract program for handling basic EHR operations. The smart-contract program contains two layers of electronic health records (EHR). The first layer keeps crucial patient information (e.g., social security identification, patient name, address, blood group, drug allergy, and medical condition), while the second layer retains patient visit information (e.g., visit date, present illness, chief complaints, diagnostic results, ICD10). Blockchain technologies are evaluated under several criteria: performance, maturity, node management, development fulfillment, and security. Four blockchains are Ethereum [30], Hyperledger [30], EOS [31], and Tendermint. Each blockchain technology has different implementation and development languages. For example, Ethereum uses Solidity language for implementation, and Tendermint uses Golang programming language. Although the different implementation of each Blockchain technology, the experimental of this research have created a common application program interface (API) to perform standard measurement for basic EHR operations (e.g., read patient visit history, create patient registration and visiting record, and update patient information).

Table 1 illustrates the blockchain comparison, including the advantage and drawbacks that are appropriate for managing EHR.

Another supporting infrastructure of the telehealth platform is the big data technology [32]. With multiple data sources from wearable and health IoT devices, enormous data steaming from patients' biological sensors are loaded into the telehealth platform for health tracking and monitoring purpose. Typically, wearable and health IoT devices periodically send health data every interval period from all patients simultaneously. Thus, streaming processing (e.g., Apache Kafka) handles real-time data streaming with high throughput messages before loading data into big data technology [33]. Explicitly, the big data technology can store structured and unstructured data (e.g., ECG image) with redundancy and scalable storage infrastructure. Thus, both patients and telehealth participants can access the health tracking data from the big data technology.

This research has evaluated which big data technologies are appropriate to keep a small data package with high volume into the storage. For example, the vital sign information, including blood pressure, temperature, and heart rate, is bundled in patients' data packages. The experimental study is a simulation generating those data packages simultaneously and putting them into big data infrastructure. The big data technologies assessment has been conducted by using the exact hardware cluster specification. There are five studies of the big data solutions for evaluation, including KUDU, HIVE, Cassandra, HBase, and Elasticsearch. The comparison is performed by writing simulation programs to insert and read data for each study simultaneously.

Table 2 shows the performance comparison for each study, including other features that should be considered during the implementation. The experimental result indicates that Elasticsearch [34] technology provides the best performance for maintaining health data for telemonitoring services.

Moreover, with advances in data analytics and machine learning, telemonitoring can capture an abnormal sign from patient health tracking information by analyzing data collections. In addition, a particular medical health record is an image output (e.g., X-ray, ECG, Ultrasound), which also resides in big data technology. With maturity in artificial intelligence technology, an outcome of data analyzing those images could predict the probability of the abnormal sign or specify the potential of the patient illness automatically with shortened periods. Once an abnormal sign has been found, the platform will notify a primary care provider to investigate further. Finally, the patient will get a notification of health concerns and make an appointment to discuss with physicians in a follow-up visit.

The teleconference infrastructure is another crucial component of the telehealth platform. So, the teleconference must provide the smoothest streaming video conference among the participants and patients. Therefore, the cloud-based infrastructure of video streaming services is recommended from a large-scale telehealth platform design. Furthermore, due to multiple concurrent sessions with real-time communication with patients, thus the teleconference infrastructure must be redundant and scalable to take care of a heavy load without delay. Optimizing adaptive bitrate streaming is necessary to adjust video resolution on demand when the teleconference is at the peak load.

**Figure 7:**
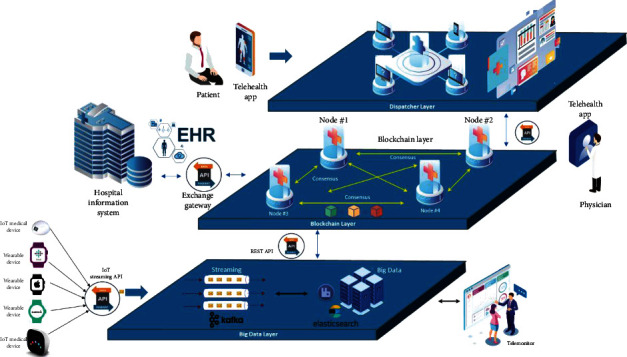
Software architecture design for a large-scale telehealth platform.

The last infrastructure component in the telehealth platform is the data exchange gateway. Although the blockchain EHR is used to synchronize the health record among the healthcare providers, there is a need to access data from telehealth platforms to participate directly. For example, participants usually demand summarized information about patient treatments, cost of healthcare services, reimbursement status, and other necessary information to operate with the telehealth platform. The patient also needs to import and export health information from the platform using this exchange gateway.  Figure 7 shows another perspective of the three layers of software architecture design for the telehealth platform, including intelligence call-dispatcher, EHR blockchain, and big data for telemonitoring.

In conclusion, the critical success factor of a large-scale telehealth platform design is the efficiency and effectiveness of serving patients in real-time. The pass-through rate of services is the primary interest when patient's services reach the maximum available resources. Therefore, the large-scale telehealth platform must design to accommodate the fluctuation of demands (patient volume) and supplies (available healthcare professionals) at a particular period.

In addition, a good design of the telehealth platform should welcome participants to join and access the platform easily across the hospital boundary or independent participants. Consequently, the system must keep track availability status of each participant in any role accurately when a participant joins the telehealth platforms and is available to serve the patients or when a participant is still busy actively serving other patients. For example, as soon as a healthcare professional is accessible around the globe, the system must assign waiting patients right away. In addition, when the demand side is high, the platform can alert an inactive healthcare member to immediately join and access the telehealth platform to distribute the workload until the demand goes down.

## 7. Telehealth Security Design

**Figure 8:**
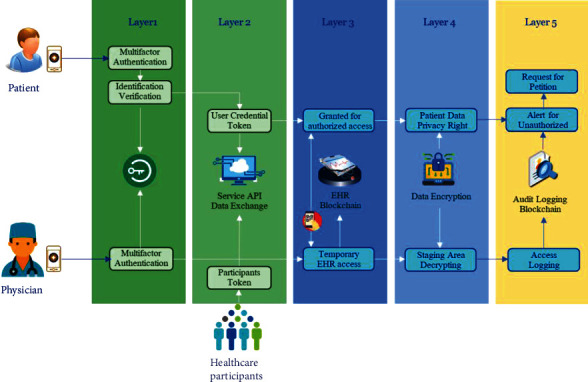
Security design for a large-scale telehealth platform.

The security design in the telehealth platform is also a crucial element. The security is embedded in multiple layers on the telehealth platform shown in Figure 8. The front-end layer is the patient authentication and identification verification, which proves that the patient is the same person within their legal identification card. The proof is used to validate the eligibility from health insurance carriers and claim reimbursement. Typically, this security layer ensures that the business knew its customer well before letting them onboard. The process involves the relationship with identity fraud and antimoney laundering controls as well as related regulatory standards, make Know Your Customer (KYC) [35]. After passing the KYC process, multifactor authentication (MFA) [36] is an authentication method that requires the user to provide two or more verification factors to gain access to the application of the telehealth platform. Rather than just asking for a username and password, MFA requires one or more additional verification factors, which decreases the likelihood of a successful cyberattack.

The second layer is the accessing security to API services and data exchange gateway of the telehealth platform. The participants are required to collaborate profoundly with the telehealth platform to coordinate interprocess operations among healthcare participants. The patient and physician application also need access to the telehealth platform by using API services. The security design of this layer usually applies token-based authentication from user login credentials [37]. Token-based authentication is a protocol that allows user's applications to verify their identity and receive a unique access token in return. During the token's lifetime, the user application access the API services that the token has been issued for, rather than reenter credentials each time accessing the API services or any resource protected from the platform. Token-based authentication is different from traditional password-based or server-based authentication techniques. Tokens offer a second layer of security, and administrators have detailed control over each action and transaction. The platform can also define a limitation in the number of access requests per minute. If the access request exceeds the threshold boundary, the platform will block the user application to access and alert the administrators to investigate.

The third layer is the authorization mechanism of the electronic health record (EHR), which is limited to the patient and relevant healthcare professionals only. The authorization mechanism controls grants or revokes a right to access EHR in the blockchain [21]. EHR accessing by relevant healthcare professionals is the most challenging design in data privacy control, while the patient must grant the authorization and limit the access to EHR. A digital handshaking approach is a solution to protect patients' EHR records from participants or healthcare professionals who are not involved with the patient case. With exchange credentials, the approach grants access to patient's EHR records to particular healthcare professionals within a certain period. Implementing a handshaking mechanism is simply using a QR code from the patent's application for healthcare processional to scan and get authorized to access the patient EHR. A patient can also specify to grant a series of access authorization to healthcare professionals that require access to their EHR in a specified hospital.

The fourth security layer is the cryptography in the telehealth platform. Cryptography applies to almost every data element, especially the electronic medical record on the blockchain. The practice of cryptography has also ensured the security of protected EHR records and relevant information. Specifically, encryption has enhanced the security of EHRs during the access and exchange of health information. In the healthcare industry, many regulations and acts of the platform need to comply with the criteria for organizations when creating, receiving, maintaining, or transmitting protected health information. Encryption and decryption methods are also successful when used to secure access through the patient health record [38]. However, when patients grant access authorization to the relevant healthcare professionals, sharing ciphertext is a hurdle to design. A straightforward solution is to duplicate the patient EHR record into the staging area by decrypting a ciphertext using the patient key into plain text and then encrypt with the healthcare professional key putting in the staging area. Periodically, the encrypted EHR in the staging area is cleaned for the specific given period. After those periods expire, the healthcare professionals cannot see patient EHR, except asking for authorization again. Cryptography is not limited to apply only EHR in the blockchain but also use the structure and nonstructured data in the big data technology. Therefore, although the protection implementation approach may design differently, the purpose of encrypting is the same.

The last security layer is the user access audit logs. Audit logs are used to capture the actions of healthcare participants of data sharing in the telehealth platform for auditors to check compliance with privacy policies. Access logs ensure that there is no violation by privileged users to access sharing patient data without authorization. Typically, audit logs are kept in a system file or a database. However, the audit logs can be tampered by high privileged users. The design solution to this problem is to keep the audit logs in the blockchain. The blockchain establishes transparency of audibility and traceability. Therefore, audit log transactions in a blockchain provide sufficient appropriate audit evidence related to the nature of the transaction [39]. With blockchain technology, it is almost impossible for any high privileged user account to alter the audit logs. Therefore, the design on this security layer enables audit access logs to be immutable and ensures genuineness, which can be used in compliant audit and prosecution pleading. A feature of using blockchain technology to real-time monitor authorized access is the event-driven message, which is messages generated at the time of occurrences such as unauthorized access of patient medical records including pictures, audio, or videos.

The mechanism offers a message alert to a patient when someone accesses their medical records. The patient can review the access logs and realize the granted authorization of those persons. If the patients have found unauthorized access to their medical records, they can request a petition for investigation or prosecution pleading to those persons. The security architecture and communication solutions for the telehealth platform aim to protect the data privacy of patients.

Please note that the security design in the paper is designated for the telehealth platform. Other security elements involving hardware and infrastructure design are not in the scope of the paper. Infrastructure security is a process of protecting the underlying networking by installing preventative measures to deny unauthorized access, modification, deletion, and theft of resources and data. These security measures can include access control, firewalls, virtual private networks (VPN), behavioral analytics, intrusion prevention systems, and wireless security.

## 8. Conclusion

The large scale of the telehealth platform is the future digital health services for all citizens in every country. In China, a telehealth platform or internet hospital currently has more than a thousand participants joining the internet hospital to serve a million patients per month [40, 41]. Inevitably, the direction of healthcare service providers and participants now transforms into digital services along with the advance of technology. The paper illustrates the design blueprint of a large-scale telehealth platform design to manage the smooth patient journey, potential digital healthcare functionality for new generation patients, and infrastructure design and security design as a guideline for an enterprise that plans to invest and implement the future telehealth platform. The design is based on optimization of sharing economic approach, which allows all participants, hospitals, healthcare institutions, independent health professionals, pharmacies, delivery partnerships, and other healthcare-related business participants, to join the platform to serve an unlimited number of patients simultaneously. The heart of design also focuses on creating a lean process and integrated workflow between healthcare participants, which is essential for healthcare institutions to join the platform without significantly modifying their internal system. Thus, the telehealth platform is the one-stop service application for the patient that can reach every isolated healthcare provider in one place and rearrange service orchestration by their preference. With all participant collaboration under the telehealth platform, the paper's patient benefits are a crucial success. Ultimately, the patient can reduce the cost of travel and healthcare services with more convenience and accessibility, while the healthcare provider can leverage the resources and service bottleneck to distribute the workload remotely based on available resources. In addition, the health insurance carriers decrease their claim expenses and offer a new product to match a niche market segmentation. Finally, telehealth platforms establish a completed digital healthcare service and new ecosystem that provides better care for every patient worldwide.

## Figures and Tables

**Table 1:** Blockchain technology comparisons for telehealth platform.

Measurement	Ethereum	Hyperledger	EOS	Tendermint
*Blockchain performance*				
(1) Number of transactions per second (TPS)	15-30	300	500	2,002
(2) Data reading response time of 1,000 records under 10 concurrent sessions	3.72 min	1333 ms	1 ms	3 ms
(3) Insert of one record response time under 10 concurrent sessions with duplication check	209 ms	235 ms	9662 ms	3 ms
(4) Update data in one record response time under 10 concurrent sessions	208 ms	544 ms	260 ms	2 ms
*Blockchain mutuality*				
(5) Mutuality level (5 years)	Y	Y	N	Y
(6) Live application in health technology	Y	Y	Y	Y
*Node management*				
(7) Fault tolerant (nodes)	2	3 (orderer)	(ratio 2/3)	(ratio 2/3)
(8) Restructure blockchain (add nodes)	N	Y	N	N
*Application fulfillment*				
(9) Smart contract language supports	Solidity	Go	C++	Go, Js
(10) Transaction management	N	Y	N	N
(11) Cryptocurrency account balance for trading	Y	N	Y	Y
(12) Access data from smart contract with ranges	N	Y	Y	N
(13) Creating index on data from smart contract	N	N	Y	Y
*Blockchain security*				
(14) Authentication and account management	Y	Y	Y	Y
(15) Encryption data in the smart contract	N	Partial	N	N
(16) Access control on smart contract	N	Y	N	N

**Table 2:** Big data technology comparisons for IoT streaming in telemonitoring.

	Study #1	Study #2	Study #3	Study #4	Study #5
Big data technology	KUDU	Hive	Cassandra	HBase	Elasticsearch
Big data Interface	Library	JDBC	Library	Library	RapidMQ
(1) Insert data^∗^					
Insert 100 b/100k rows	1.54	13,207.80	1.11	1.22	0.34
Insert 1 kb/100k rows	1.70	No response	No response	1.31	0.75
Insert 10 kb/100k rows	2.73	No response	No response	2.73	7.30
Insert 100 kb/100k rows	17.72675	No response	No response	23.4235	13.33
(2) Select data^∗^					
Select 1 row	800.00	19,709.20	No response	1,000.00	230
Select 5 rows	800.00	21,882.20	No response	No response	450
(3) Architecture	Master/slave	Master/slave	Multimaster	Master/slave	Master/slave
(4) File structure	Distributed DB	Hadoop	Distributed DB	Hadoop	Database
(5) Storage fault tolerant	Multicopies	Multicopies	Multicopies	Multicopies	Multicopies
(6) Large volume data access for analytics	Average	Best	Worse	Worse	Average
(7) Access data by key	No	No	Yes	No	No
(8) Built-in visualization	No	No	No	No	Best
(9) Min. server required	3	5	3	3	3
(10) Security					
Client-node encryption	TLS/SSL		TLS/SSL	N/A	TLS/SSL
Web UI encryption	TLS/SSL		N/A	N/A	
Peer node authentication	N/A		N/A	N/A	
User authentication	Kerberos		Internal	Kerberos	
Authentication tokens	Y		N/A	Kerberos	

^∗^A unit of measurement is a response time (millisecond) per 1 transaction.
